# Common uropathogens among diabetic patients with urinary tract infection at Jinja Regional Referral Hospital, Uganda

**DOI:** 10.4102/ajlm.v7i1.621

**Published:** 2017-02-09

**Authors:** Barbara I. Nabaigwa, Bashir Mwambi, John Okiria, Caesar Oyet

**Affiliations:** 1 International Health Sciences University, Kampala, Uganda

## Abstract

Between June 2015 and October 2015, 159 mid-stream urine samples from diabetic patients were cultured. The prevalence of urinary tract infection was high at 22% and women were more affected compared with men (*P* = 0.017). Factors associated with urinary tract infection in these patients were age, sex and high blood glucose levels. Diabetic patients should be screened periodically for urinary tract infection.

## Introduction

Urinary tract infection (UTI) is common among both adults and children. According to Tandogdu and Wagenlehner,^1^ prevalence of UTI varies greatly worldwide from 0.7% to 20%. Patients with diabetes mellitus are highly susceptible to UTI,^2,3^ and up to 35% of diabetic patients experience a UTI. A number of factors predispose patients with diabetes to UTI. These factors include weak host immune systems with impaired neutrophil function, depressed T-cell-mediated immune response, decreased production of prostaglandin E, thromboxane B2 and leukotriene B4^4^ and depressed antioxidant systems,^5^ all of which expose such patients to infection. Urinary incontinence due to disorders of the autonomic nervous system leads to incomplete bladder emptying, which in turn allows uropathogens to colonise and invade the urogenital niches.^4^ Presence of glucose in urine of the diabetic patients, coupled with poor metabolic control, provides a conducive environment for pathogenic bacteria to flourish and cause UTIs.^6^

Several uropathogens have been implicated in diabetic patient infections. The most common uropathogens isolated from diabetic patients are *Escherichia coli, Klebsiella* spp., *Staphylococcus aureus* and *Candida albicans*.^7^

This study was conducted to establish which UTI aetiological agents are most common among diabetic patients attending the diabetes clinic at Jinja Regional Referral Hospital. Risk factors for UTIs by these pathogens were also evaluated.

## Methods

### Ethical considerations

This study was approved by the Institutional Review Board of the International Health Sciences University in Kampala, Uganda (approval number: UGS 420-2016). Research staff explained the study to eligible participants. Patients who agreed to participate in the study signed consent forms.

### Study design

This was a cross-sectional study conducted between June 2015 and October 2015. A total of 210 diabetic men and women aged between 18 to 70 years who presented with a medical history of painful urination, urinary incontinence and lower abdominal pain at the time of the study and who provided informed consent to participate were recruited by systematic sampling. Upon arrival, patients were requested to pick a card from a box containing three numbers: 1, 2 or 3. Every fourth patient was enrolled after a patient picked a card with the number 2. Participants were instructed to collect about 20 mL of mid-stream urine into a pre-labelled sterile screw-capped, graduated, wide-mouth plastic container. Urine specimens were transported to the laboratory at 4°C within two hours of collection.

All the specimens were subjected to a leukocyte esterase and nitrite test using a dipstick rapid test from Cypress Diagnostics (Langdorp, Belgium). Urine specimens positive for nitrites or leukocyte esterase were subjected to bacterial culture.

Using a 0.002 mL, calibrated wire loop, urine specimens positive for nitrites or leukocytes were aseptically inoculated on cystine-lactose-electrolyte deficient medium (Oxoid Ltd, Basingstoke, Hampshire, United Kingdom) and blood agar (Oxoid Ltd, Basingstoke, Hampshire, United Kingdom), then incubated aerobically overnight at 37°C. Pure growth with > 10^5^ colony-forming units (CFU) per millilitre of urine was considered positive. Mixed cultures and growth with < 10^5^ CFU were considered negative. Standard reference strains, *Staphylococcus aureus* (ATCC25923), *Escherichia coli* (ATCC25922) and *Pseudomonas aeruginosa* (ATCC 27853) were used as controls for testing the quality of culture media.

A non-fasting 5-mL blood specimen was collected from each participant in oxalate/fluoride vacutainers (Becton, Dickinson and Company, Franklin Lakes, New Jersey, United States). Each specimen was analysed on a Cobas Integra 400 analyzer (Roche Diagnostics, Indianapolis, Indiana, United States) for blood glucose level. A blood glucose level of > 7.8 mmol/L was considered to be hyperglycaemic.^8^

### Data management and analysis

Data were analysed using STATA, version 11 (StataCorp, LLC, College Station, Texas, United States). A 95% confidence level was used for the analyses. Multivariate logistic regression was used to evaluate associations of urinary tract infection with age group and blood glucose. The Chi-square test was used to compare occurrence of urinary tract infection between men and women. *P*-values of less than 0.05 were considered statistically significant.

## Results

A total of 210 diabetic patients consented and were recruited, of whom 122 (76.7%) were women. The rapid dipstick test showed that a total of 159 (75.7%) of the urine specimens were positive for either nitrites, leukocytes or both; these were cultured and included in the analysis. Thirty-five of the 159 cultures were positive for bacterial growth (> 10^5^ CFU/mL of urine) giving the prevalence of UTI at 22.0%. Of the 122 female participants, a total of 29 (23.8%) had a UTI compared with 14 (15.9%) of 88 men (*P* = 0.017). Also, 22 out of the 35 positive culture were from patients aged over 50 years (*P* = 0.003) (Table 1), and 28 of 35 (80%) uropathogens were isolated from participants with hyperglycaemia (*P* = 0.0026). Only 7 of the 35 (20%) isolates were obtained from participants with a normal glucose level, whereas no isolate was obtained from participants with low fasting blood glucose (*P* < 0.0001). Eighteen of the 35 (51.4%) isolates were *Staphylococcus saprophyticus*, 12 (34.3%) were *Escherichia coli*, 3 (8.6 %) were *Klebsiella* spp., 1 (2.85%) was *Citrobacter* spp. and 1 (2.85%) were enterococci.

**TABLE 1:** Distribution of isolates by participant age (*n* = 159).

Age group (years)	18–30	31–40	41–50	> 50
No growth (%)	9 (69.23)	18 (85.71)	40 (86.96)	57 (72.15)
*Staphylococcus saprophyticus* (%)	3 (23.08)	3 (14.29)	5 (10.87)	7 (8.86)
*Escherichia coli* (%)	1 (7.69)	-	1 (2.17)	10 (12.66)
*Klebsiella* spp. (%)	-	-	-	3 (3.80)
Others (%; *Citrobacter*, enterococci)	-	-	-	2 (2.54)

**Total**	**13**	**21**	**46**	**79**

## Discussion

Urinary tract infection, defined as the presence of > 10^5^ CFU of bacteria per mL of fresh urine,^9^ is a common type of infection. The risk of UTI is two to three times higher among patients with diabetes than among their non-diabetic counterparts.^9^ The overall prevalence of UTI in our study was 22.0%, which is high compared to a study conducted among Romanian patients where the prevalence of UTI was 12.0%.^10^ This difference in prevalence could be due to, among other causes, variations in socioeconomic status.^2^ Diabetes management is expensive; thus, low-income countries such as Uganda are more prone to advanced effects, including UTI, compared with middle-income countries such as Romania. However, the prevalence of UTI in our study was similar to that of a study in Sudan where prevalence was 19.5%^12^ and the socio-demographic environment is similar to Uganda. These findings call for earlier interventions to control diabetes in low-income countries.

More women had UTI compared to men (29/35, *P = 0.017*). This finding agrees with observations from other studies, in which female patients had a higher risk of UTI than male patients.^1^ The cause of higher prevalence of UTI among women is thought to be due to anatomical predisposition compared with men. Women have a short and wide urethra with close proximity to the anus, allowing intestinal organisms easier access to the urethra. Another possible cause may be due to changes in the physiological environment of the vagina among diabetic women, such as decreases in normal vaginal flora and a less acidic pH of the vaginal surface.^14^ This may be exacerbated among women with poor hygiene.^18^

Our study also found that the number of UTI cases increased with increased age. Participants older than 50 years were more affected (22/35) compared with other age groups. Another study, by Wilke et al., found similar associations between age and UTI.^17^ Higher numbers of cases of UTI among older diabetic patients could be the result of decreased urinary flow, coupled with incomplete bladder emptying resulting from neuropathy, reductions in oestrogen with loss of vaginal flora among women and prostate disease in men.^17^

Our study revealed that hyperglycaemia was positively associated with UTI among diabetic patients. Up to 80% of the isolates were from participants with high blood glucose levels (> 7.8 mmol/L) compared with participants with normal glucose levels (< 7.8 mmol/L). The high UTI prevalence among hyperglycaemic patients is most likely due to poor contraction of a dysfunctional bladder leading to static urine pools; this, together with glycosuria, creates a suitable environment for bacterial growth. However, some studies have not detected associations between UTI and blood glucose level.^13^ This may be because a single blood glucose measurement may not represent glycaemic control over time, which would predispose diabetic patients to UTI.

The most common uropathogen in our study was *S. saphrophyticus*, followed by *E. coli*. This finding is not in agreement with several studies conducted on diabetic patients. A study in Bangladesh showed that bacteria of the Enterobacteriacae family, especially *E. coli* and *Klebsiella* spp., are the most common uropathogens in diabetic patients.^19^ Another study also demonstrated *E. coli* as the most common uropathogen responsible for UTI in diabetic patients.^7^ The explanation for this discrepancy may be differences in study design and sample size or the fact that *S. saprophyticus* infections often yield < 10^5^ CFU/mL of urine even in suprapubic aspirated urine samples. This would mean that in many instances growth of this organism is considered non-significant in routine urine culture performed on mid-stream urine. Our study had a smaller sample size with a higher proportion of women, who harbour *S. saprophyticus*. The small sample size could account for our low rates of *E. coli*. Another reason could be differences in the population distribution of the organisms, since most studies have been performed outside of Uganda and there is little literature on the distribution of these organisms among diabetic patients in Uganda.

### Recommendations

We recommend periodic screening of diabetic patients for UTI. A study with larger sample size and power should be conducted to evaluate the distribution of uropathogens among diabetic patients in Uganda.

### Limitations

A limitation to our study that should be considered when interpreting its results is that the sample size was small. This limited the power of the study for some analyses.

### Conclusions

There is high prevalence of UTI among diabetic patients in Uganda. Age, sex and high blood glucose were associated with UTI in this group of diabetic patients.
